# New Insights into the Antibacterial Activity of Hydroxycoumarins against *Ralstonia solanacearum*

**DOI:** 10.3390/molecules21040468

**Published:** 2016-04-08

**Authors:** Liang Yang, Wei Ding, Yuquan Xu, Dousheng Wu, Shili Li, Juanni Chen, Bing Guo

**Affiliations:** 1 Laboratory of Natural Products Pesticides, College of Plant Protection, Southwest University, Chongqing 400715, China; ylwzling@163.com (L.Y.); swudousheng@163.com (D.W.); lsl203lst@163.com (S.L.); chenhuanni521@126.com (J.C.); guobing425@163.com (B.G.); 2 Biotechnology Research Institute, The Chinese Academy of Agricultural Sciences, Beijing 100081, China; xuyuquan@caas.cn

**Keywords:** *Ralstonia solanacearum*, bacterial wilt, hydroxycoumarins, antibacterial activity, biofilm inhibition, daphnetin, esculetin

## Abstract

Coumarins are important plant-derived natural products with wide-ranging bioactivities and extensive applications. In this study, we evaluated for the first time the antibacterial activity and mechanisms of action of coumarins against the phytopathogen *Ralstonia solanacearum*, and investigated the effect of functional group substitution. We first tested the antibacterial activity of 18 plant-derived coumarins with different substitution patterns, and found that daphnetin, esculetin, xanthotol, and umbelliferone significantly inhibited the growth of *R. solanacearum*. Daphnetin showed the strongest antibacterial activity, followed by esculetin and umbelliferone, with MICs of 64, 192, and 256 mg/L, respectively, better than the archetypal coumarin with 384 mg/L. We further demonstrated that the hydroxylation of coumarins at the C-6, C-7 or C-8 position significantly enhanced the antibacterial activity against *R. solanacearum.* Transmission electron microscope (TEM) and fluorescence microscopy images showed that hydroxycoumarins may interact with the pathogen by mechanically destroying the cell membrane and inhibiting biofilm formation. The antibiofilm effect of hydroxycoumarins may relate to the repression of flagellar genes *fli*A and *flh*C. These physiological changes in *R. solanacearum* caused by hydroxycoumarins can provide information for integral pathogen control. The present findings demonstrated that hydroxycoumarins have superior antibacterial activity against the phytopathogen *R. solanacearum*, and thus have the potential to be applied for controlling plant bacterial wilt.

## 1. Introduction

Bacterial wilt, caused by *Ralstonia solanacearum*, causes destructive economic losses to important crops such as tomato, potato, tobacco, and eggplant in tropical and subtropical regions [1,2]. It is estimated that bacterial wilt generates at least $1 billion in losses each year, with an especially devastating effect on staple crops in developing countries [3]. Currently, the main methods for controlling bacterial wilt are based on integrated pest management and chemical control. However the effectiveness of these methods diminishes with long-term field application [4]. For example, the traditional chemical control induces successive occurrence of pesticide-resistant strains and raises environmental safety concerns [5,6]. Due to the limitations of current control methods, the increasing host range of *R. solanacearum* and its complex pathogenicity and range of infectious conditions, it is difficult to develop effective measures to protect plants against *R. solanacearum* [7,8,9]. This situation demands alternative agents or measures to effectively control bacterial wilt.

More recently, biological control has been considered a promising management strategy for bacterial wilt, especially using plant-derived compounds or plant extracts [10,11,12]. These compounds exhibit the ability of suppressing soil-borne pathogens and promoting plant growth [13,14]. As previously reported, bacterial wilt was suppressed by lansiumamide B [10], flavonoids [11], palmarosa (*Cymbopogon martini*), lemongrass (*C. citratus*), and eucalyptus (*Eucalyptus globulus*) oils [14], and methyl gallate [15]. However, few studies have focused on the mechanism of the antibacterial action of these compounds against plant pathogenic bacteria, especially *R. solanacearum*. Moreover, coumarins, a group of promising biocontrol compounds, have not yet been tested against *R. solanacearum*.

Coumarins are natural compounds produced by a wide range of plant sources. Their structures comprise fused benzene and α-pyrone rings. Recently, coumarins have attracted extensive research interests due to their biological activities. Which include antibacterial, antifungal, anticoagulant, antioxidant, anticancer, and anti-inflammatory properties [16,17]. Studies have reported that coumarins exhibited strong antibacterial activity on both Gram-positive and Gram-negative bacteria, especially against Gram-negative bacteria, by damaging the cell membrane [17,18,19]. The advantages of coumarins as promising antibacterial compounds include: (1) a board spectrum of antibacterial activity; (2) they are secreted by plants as phytoalexins to defend against attacks from pathogens; (3) they are environmentally friendly and not susceptible to develop bacteria resistance. Our previous studies also indicated that plant-derived compounds like coumarin, protocatechuic aldehyde, resveratrol, and carvacrol had antibacterial activity against *R. solanacearum*. Furthermore, coumarins can serve as scaffolds for advanced design and synthesis of more active derivatives [20]. Taken together, these facts highlight the value of investigating the antibacterial activity of coumarins against *R. solanacearum*. Consequently, in this study, we tested the antibacterial activity of 18 coumarins against *R. solanacearum*, and found that hydroxycoumarins such as daphnetin and esculetin showed the strongest bioactivity. We further focused on the hydroxycoumarins to investigate the antibacterial mechanism of action, including biofilm inhibition and regulation of some virulence-associated genes of *R. solanacearum*. Our findings show that hydroxycoumarins have great potential as effective botanical bactericides for controlling bacterial wilt.

## 2. Results and Discussion

### 2.1. Antibacterial Activity of Screened Coumarins against R. solanacearum

**Figure 1:**
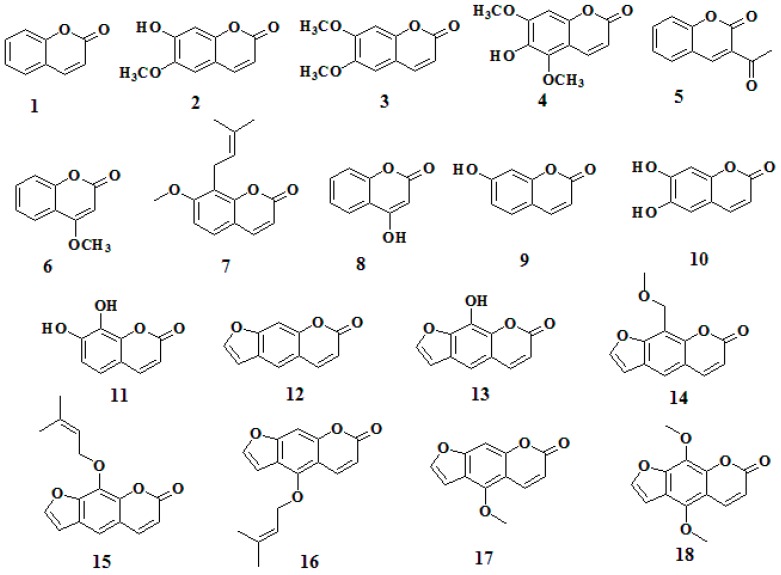
The chemical structures of the studied coumarins.

Initially, we evaluated the antibacterial activity of plant-derived compounds against *R. solanacearum*. Compared with thiodiazole copper treatment, umbelliferone, coumarin, and tea polyphenols exhibited strong antibacterial activity (Table S1). Further, in order to determine whether coumarin derivatives had antibacterial activity on *R. solanacearum*, we evaluated the antibacterial activity on *R. solanacearum* exposed to 18 coumarins at two dosage levels. The results showed that seven coumarins had strong antibacterial effects against *R. solanacearum* with an antibacterial rate over 50% after 24 h treatment at a dosage of 100 mg/L. Compared with thiodiazole copper treatment, among the screened coumarins, daphnetin (7,8-dihydroxycoumarin, **11**, Figure 1) showed the highest antibacterial activity, followed by xanthotol (**13**), and esculetin (6,7-dihydroxycoumarin, **10**) with antibacterial activity rates of 97.43%, 80.12%, and 71.44%, respectively (Table 1), significantly higher than the thiodiazole copper treatment with an antibacterial rate of 63.6%. Interestingly, hydroxyl substitution, especially on the C-6, C-7, or C-8 sites, seemed to enhance the antibacterial activity of coumarins against *R. solanacearum*. Thus we chose hydroxycoumarins, *i.e.*, umbelliferone, esculetin and daphnetin, for further investigation of the detailed antibacterial mechanism.

### 2.2. MIC and MBC of Hydroxycoumarins against R. solanacearum

The minimum inhibitory concentration (MIC) and minimum bactericidal concentration (MBC) of hydroxycoumarins against *R. solanacearum* were measured using the typical microdilution method. As shown in Table 2, daphnetin was the most effective compound against *R. solanacearum*, followed by esculetin, umbelliferone, and coumarin. The MIC of daphnetin was 64 mg/L, much lower than the value of the parent compound coumarin (384 mg/L), which indicated the inhibitory efficiency of daphnetin is 6-fold that of coumarin. The MICs of umbelliferone and esculetin were 256 and 192 mg/L, respectively. The minimum bactericidal concentrations (MBCs) of coumarins on *R. solanacearum* were defined as the lowest concentration of coumarins that prevent the growth of bacteria after sub-culture on agar media. As shown in Table 2, the MBCs of coumarin, umbelliferone, esculetin and daphnetin against *R. solanacearum* were 512, 384, 192, 64 mg/L, respectively.

### 2.3. Hydroxycoumarins Inhibit the Growth of R. solanacearum

**Figure 2:**
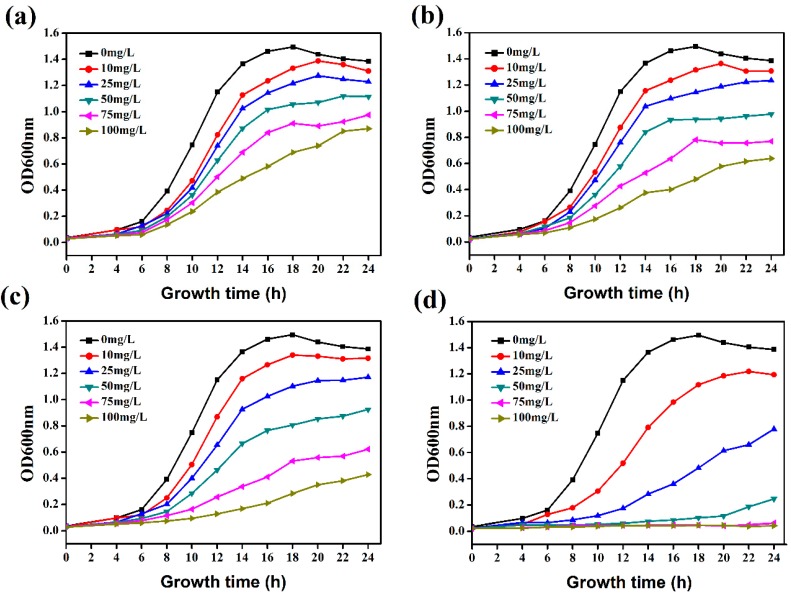
The effect of coumarins at concentrations ranging from 10 to 100 mg/L on the growth curves of *R. solanacearum*: (**a**) coumarin; (**b**) umbelliferone; (**c**) esculetin; (**d**) daphnetin.

To further investigate the inhibitory effect of hydroxycoumarins and evaluate whether hydroxyl substitution enhances the antibacterial activity, we examined the growth curve of *R. solanacearum* exposed to the screened hydroxycoumarins like umbelliferone, esculetin, and daphnetin at concentrations ranging from 10 to 100 mg/L, and used the archetypal coumarin as the positive control. As shown in Figure 2, hydroxycoumarins, especially daphnetin and esculetin, siginificantly inhibited the growth of *R. solanacearum* through all the concentration range of 10 to 100 mg/L. Daphnetin almost completely stopped the bacterial growth at the concentration of 50–100 mg/L after 24 h incubation (Figure 2d). The growth of *R. solanacearum* was also inhibited by umbelliferone, esculetin (Figure 2a–c, and the antibacterial activity of hydroxycoumarins against *R. solanaccearum* increased with dosage. Further, to evaluate the quantitative correlation between the concentration and the antibacterial activity of coumarins against *R. solanacearum*, we simulated the linear relationship between the logarithmic value of coumarin concentration and the probability value of corrected antibacterial rate based on a turbidimeter test. After 12 and 24 h incubation, the growth of *R. solanacearum* entered the logarithmic phase and stable phase, respectively. As shown in Table 3, after 24 h incubation, the IC_50_ values of daphnetin and esculetin were 23.98 and 67.85 mg/L, respectively.

In summary, we can conclude that coumarins with hydroxylation in the position C-7 (umbelliferone) have strong antibacterial activity. An additional hydroxylation in position C-6 (esculetin) enhances the antibacterial activity, while an even greater improvement results from dihydroxylation in positions C-7 and C-8 (daphnetin). Coumarins have been proven to have multiple substitution sites, and different substitutions in these sites significantly affect the biological activity of coumarins [16,21,22]. Previous research found that coumarins with OH groups at C-7 or C-8 were better antibacterial compounds than the model molecules [23,24]. Daphnetin (7,8-dihydroxycoumarin) was the most active compound against the tested bacteria among 20 coumarins extract from Mexican tarragon or purchased [17]. In this case, it is probable that the two adjacent hydroxyl groups substituted on the C-7 and C-8 of the coumarin skeleton electronically activate the aromatic ring and increase the hydrophobic and lipophilicity, which consequently promote the combination of the hydroxycoumarins with the membrane of bacteria and enhance the activity against bacterial strains [17,25]. Other substituents were also proved to change the structure of compounds and affect their antibacterial activity. Four example, 2-aryl-4,5-dihydrothiazole analogues exhibited strong bioactivity on *R. solanacearum* unless a 2′-hydroxyl group was introduced on the 2-aryl substituent of the compound [26].

### 2.4. Bacterial Morphological Change by TEM

**Figure 3:**
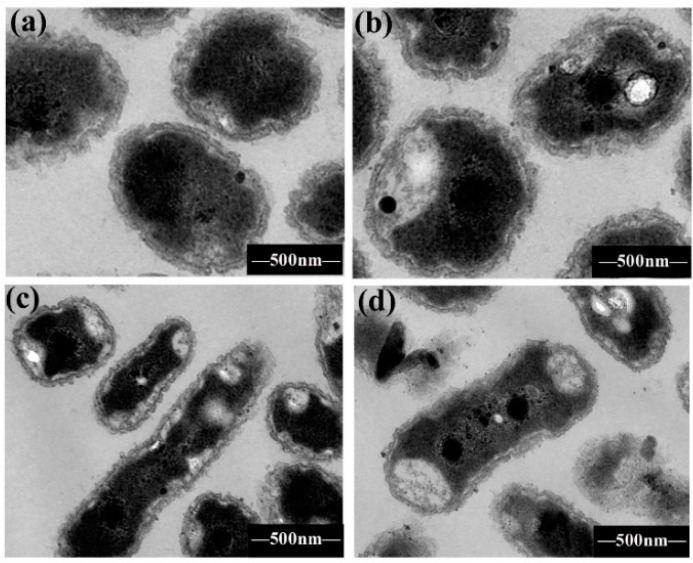
TEM images of *R. solanacearum* cells treated with (**a**) DMSO; (**b**) umbelliferone; (**c**) esculetin; and (**d**) daphnetin, respectively. Overnight cultured bacterial suspension was diluted into B medium supplemented with coumarins (50 mg/L umbelliferone, 50 mg/L esculetin and 25 mg/L daphnetin) and incubated at 30 °C for 12 h.

In order to further investigate the mechanism of antibacterial activity of coumarins, the cell morphology of *R. solanacearum* was monitored using TEM after treatment with hydroxycoumarins. The TEM images of *R. solanacearum* were taken after the addition of 50 mg/L hydroxycoumarins (umbelliferone, esculetin) and 25 mg/L daphnetin. According to Figure 3, daphnetin and esculetin induced irreversible damage to the cell membrane of *R. solanacearum*, producing irregular hollows in the cells. These results were in agreement with the observed antibacterial activity of hydroxycoumarins against *R. solanacearum*. However, the insignificant change caused by umbelliferone indicated other mechanisms leading to the bacterial death. Daphnetin and esculetin are widely distributed hydroxycoumarins throughout the plant kingdom with several promising biological activities, such as antibacterial and antioxidant, and phenolic activities [17,27]. Previous studies have identified several plant phenolic derivatives as promising antibacterial inhibitors of *Escherichia coli*, *Hafnia alvei*, and *Xylella fastidiosa* [28,29]. This is consistent with our finding that hydroxycoumarins have strong antibacterial activity against *R. solanacearum*. The antibacterial mechanism of phenolic compounds like carvacrol and thymol was attributed to membrane potential changes [30]. In agreement with these observations, our results also showed that daphnetin and esculetin induced the irreversible damage to the cell membrane of *R. solanacearum*. Literature reports indicate that activation of phenolic metabolites and phytoalexins could be expressed against pathogens which is considered to strongly limit the spread of invading pathogens [31]. Our findings suggest that hydroxycoumarins have superior antibacterial activity against the phytopathogen *R. solanacearum*, as the antibacterial activity of daphnetin and esculetin were significantly higher than that caused by thiodiazole copper treatment. Hydroxycoumarins had no effect on the germination of tobacco seed (Figure S2), and have low cytotoxicity on human cells [32], so the combination of antibacterial activity and low the cytotoxicity of hydroxycoumarins make them potentially useful agents for controlling plant bacterial wilt.

### 2.5. Hydroxycoumarins Reduce Biofilm Formation of R. solanacearum

Like many plant pathogenic bacteria, *R. solanacearum* forms biofilm-like aggregations on host plant roots, contributing to the bacterial invasion and infection [33]. Previous studies have proved that coumarins like scopoletin, umbelliferone, esculetin, and daphnetin were phytoalexins secreted by plants to protect themselves from the attack of pathogenic bacteria [34,35]. Meanwhile, coumarins play an important role in the chemical defense strategy of plants [36]. We thus speculated that hydroxycoumarins were plant-microorganism interaction factors between plant and *R. solanacearum*, and hydroxycoumarins may have effects on biofilm formation.

**Figure 4:**
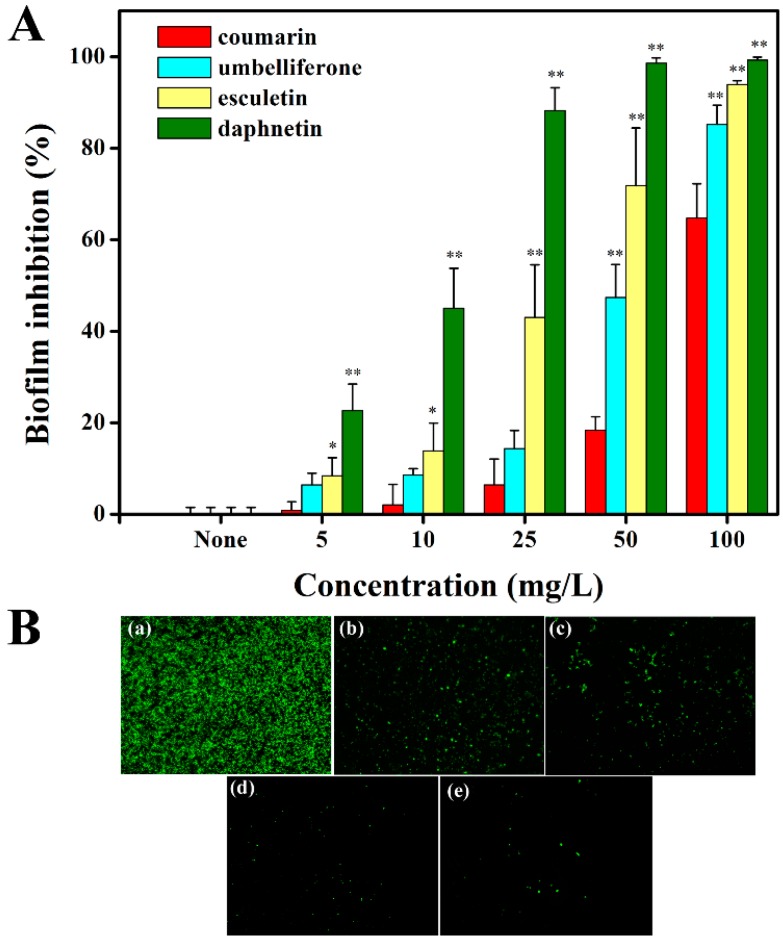
Effects of coumarins on biofilm formation of *R. solanacearum*. Biofilm inhibition (%) was quantified after treatment with different concentrations of coumarins at 30 °C for 24 h in the 96-well plates (**A**); (* indicated *p* < 0.05, ** indicated *p* < 0.01). Fluorescence microscope imaging of biofilm formation of *R. solanacearum* using FITC-ConA tagged with extracellular polysaccharide in 24-well polystyrene microtiter plate (**B**); Fluorescence microscope imaging of *R. solanacearum* with (**a**) 50 mg/L DMSO (control treatment); (**b**) coumarin (50 mg/L); (**c**) umbelliferone (50 mg/L); (**d**) esculetin (50 mg/L); (**e**) daphnetin (50 mg/L).

As shown in Figure 4a, the hydroxycoumarins significantly reduced the biofilm formation of *R. solanacearum*, especially esculetin and daphnetin. Specifically, daphnetin reduced biofilm formation by 99.22% at a concentration of 100 mg/L compared with 64.66% by coumarin treatment. Esculetin and umbelliferone also significantly reduced the biofilm formation at the concentration of 100 mg/L with inhibitory rates of 93.90% and 85.20%, respectively. The results showed that daphnetin exhibited the highest inhibitory ability on *R. solanacearum* biofilm formation, followed by esculetin, umbelliferone, and coumarin. The inhibitory activity of the coumarins was concentration-dependent. The motility of *R. solanacearum* plays an important role in biofilm formation, therefore the swimming motility under coumarin treatment was also investigated. The results showed that hydroxycouamrins like esculetin and daphnetin significantly repressed the swimming motility of *R. solanacearum* at a concentration of 50 mg/L (Figure S1).

To further validate the effect of hydroxycoumarins on *R. solanacearum* biofilm formation, the extracellular polysaccharide (EPS) involved in biofilm formation was measured in 24-well polystyrene microtiter plates with or without coumarins, using FITC-ConA as a tag which can be excited at wavelength of 488 nm (Ar laser) to generate a bright green fluorescence [37]. Daphnetin and esculetin dramatically reduced the biofilm formation compared with control treatment (Figure 4b). Daphnetin almost completely inhibited the biofilm formation (Figure 4e). The results agreed with the biofilm formation assay.

### 2.6. Hydroxycoumarins Repress Virulence-Associated Genes of R. solanacearum

**Figure 5:**
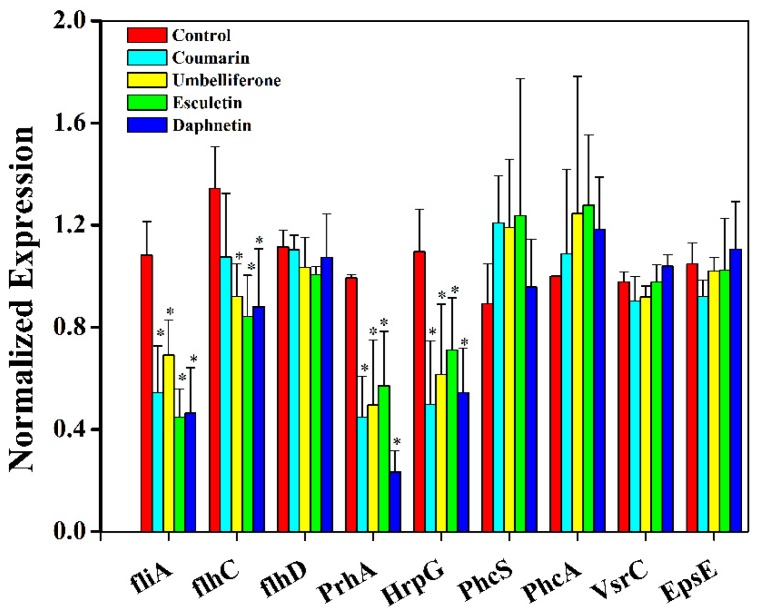
Expression of some virulence-associated genes of *R. solanacearum* were quantified by qRT-PCR treated with or without coumarins. *R. solanacearum* was cultured in B medium treated with DMSO or the coumarins using the IC_50_ concentration (coumarin 58.09 mg/L, umbelliferone 37.15 mg/L, esculetin 24.15 mg/L and daphnetin 8.73 mg/L). *Ser*C was used as the reference gene to normalize the gene expression using the ∆∆Cq method. The results reflect three biological replicates and error bars indicate the standard deviation. (* indicated *p* < 0.05)

Thanks to our increasing knowledge about *R. solanacearum*, the virulence-associated genes were comprehensively and thoroughly studied in previous studies [38,39]. For example, the regulating and structural flagellar genes, *fli*A, *flh*C and *flhD*, participate in the regulation of swimming motility of *R. solanacearum* [40]. In a preliminary study, we found that hydroxycoumarins, especially daphnetin, repressed the swimming motility and reduced the biofilm formation. In order to investigate the molecular mechanism responsible for swimming motility and biofilm inhibition, we measured the expression of the main virulence-associated genes in *R. solanacearum* cells treated or not with coumarins using qRT-PCR. The results indicated that the expression of *fli*A and *flh*C was significantly repressed by umbelliferone, esculetin, and daphnetin (Figure 5), but the hydroxycoumarins did not have effect on *flh*D and *Vsr*C which also contribute to the swimming motility of *R. solanacearum*. This result implied that coumarins (umbelliferone, esculetin, and daphnetin) reduce biofilm formation through repressing the swimming motility by repressing some flagellar genes.

In addition, coumarins had no effect on the expression of *Eps*E, *Phc*A, *Vsr*C, and *Phc*S. Interestingly, coumarins significantly repressed the expression of *Prh*A and *Hrp*G, which are the main regulators of the type III secretion system (T3SS) of *R. solanacearum*. The result indicated that hydroxycoumarins like daphnetin, esculetin, and umbelliferone have the potential to be the T3SS inhibitors. The regulation pathway of hydroxycoumarins on T3SS of *R. solanacearum* needs further investigation.

## 3. Experimental Section

### 3.1. Bacterial Strains and Coumarins

The *R. solanacearum* (phylotype I, race1, biovar 3) was used throughout the study [41]. The experiments were conducted at 30 °C in rich B medium [42]. All coumarins (Table 1) were purchased from Shanghai Yuanye Bio-Technology Co., Ltd. (Shanghai, China) and dissolved in dimethyl sulfoxide (DMSO). The final concentration of coumarins in DMSO was 10 mg/mL.

### 3.2. Antibacterial Biological Assay to Screen Active Compounds

The antibacterial activity of coumarins against *R. solanacearum* was evaluated by the turbidimeter test method with minor modifications [43]. Briefly, 50 µL overnight-cultured bacterial suspension adjusted to OD_600_ = 1.0 was inoculated in 10 mL rich B medium supplemented with coumarins to generate concentrations of 10 mg/L or 100 mg/L. DMSO (100 µL) was used as control treatment. The flasks were kept rotating on a shaker at 180 rpm and 30 °C for 24 h. The antibacterial rate of *R. solanacearum* supplemented with coumarins were calculated according to the following equation:

(1) $$\text{Antibacterial rate}\left(\text{\%}\right)=\frac{A_{0}-A_{1}}{A_{0}}\times 100\%$$

where A_0_: Corrected OD_600_ values of the control treatment; A_1_: Corrected OD_600_ values of the coumarin treatment. All assays were carried out at least three times in biological repeats.

### 3.3. Determination of MIC and MBC

The bacteriostatic and bactericidal activities of coumarins against *R. solanacearum* were examined by the minimum inhibitory concentration (MIC) and minimum bactericidal concentration (MBC) methods. The experiments were performed in 96-well polystyrene microtiter plates with minor modifications [44,45]. Briefly, 1 µL of overnight-cultured bacterial suspension adjusted to OD_600_ = 1.0 was inoculated in 198 µL rich B medium, and supplemented with 1 µL triphenyl tetrazolium chloride (TTC) as the indicator of *R. solanacearum* growth [46]. The concentrations of coumarins were adjusted to 512, 398, 256, 192, 128, 64, 32, 16, 8, 4, 2, and 1 mg/L. The polystyrene microtiter plate was incubated without shaking for 12 h at 30 °C. TTC was. After addition of TTC and incubation, the MIC was defined as the lowest concentration of compounds at which no pink color appeared. The MBC was defined as the lowest concentration of coumarins that prevented the growth of bacteria after sub-culturing on agar media. All assays were carried out at least three times in biological repeats.

### 3.4. Bacterial Growth Curve

The growth curve of *R. solanacearum* was determined as in a previous study with minor modifications [41]. Briefly, the overnight-cultured bacterial suspension (OD_600_ ≈ 1.0) was inoculated in 25 mL rich B medium supplemented with coumarins (coumarin, umbelliferone, esculetin, daphnetin) to generate a final concentration of 10, 25, 50, 75, or 100 mg/L. The control treatment contained 100 µL DMSO. Cell density was detected by measuring the optical density (OD) at 600 nm every two hours during the 24-h cultivation. All samples were performed in triplicates and calculated to obtain an averaged value. The antibacterial rate of *R. solanacearum* supplemented with coumarins after incubated for 12 and 24 h were calculated using the equation mentioned above.

### 3.5. Biofilm Assay

The biofilm formation assay of *R. solanacearum* was performed in 96-well polystyrene microtiter plates as previously reported [40]. Briefly, 1 µL overnight-cultured bacterial suspension adjusted to OD_600_ = 1.0 was inoculated in 199 µL B medium supplemented with coumarins (coumarin, umbelliferone, esculetin, and daphnetin) to generate a final concentration of 5, 10, 25, 50, or 100 mg/L. The polystyrene microtiter plate was incubated without shaking for 24 h at 30 °C. Biofilms were stained with crystal violet and dissolved in 95% ethanol and quantified by absorbance at 530 nm (OD_530_). The experiment was performed in at least six replicate wells and average values were calculated. All assays were carried out at least three times in biological repeats.

### 3.6. Swimming Motility Assay

The swimming motility of *R. solanacearum* was measured on semi-solid motility media as previously reported [47]. Medium plates contain 1% tryptone and 0.325% ager. Coumarins (50 mg/L) were added into motility medium and DMSO was used as control treatment. An overnight *R. solanacearum* culture was adjusted to OD_600_ = 0.1 and 3 µL bacterial suspension was dropped on the plate. Motility was visualized as a white halo and measured after 1–2 days of incubation at 30 °C. All assays were carried out at least three times in biological repeats.

### 3.7. Fluorescence Microscopy Imaging

The biofilm formation of *R. solanacearum* was further investigated by fluorescence microscopy using FITC-ConA tagged with extracellular polysaccharide (EPS) [37]. Briefly, the experiment was performed in 24-well polystyrene microtiter plate with or without coumarins (50 mg/L), and incubated without shaking for 12 h at 30 °C. The biofilm formation was stained with 20 µL FITC-ConA for 30 min in the dark. Static biofilm formation was visualized by inverted fluorescence microscope (Axio Observer D1, Zeiss, Jena, Germany) using an excitation wavelength of 488 nm (Ar laser) and a 200 × objective. The biofilm formation was evaluated by the number of green points in the fluorescence microscopy images.

### 3.8. Cell Morphology Observation with TEM

To measure the morphology of *R. solanacearum* after hydroxycoumarins treatments, a transmission electron microscope (TEM) was used as previously reported with some modifications [48]. Briefly, an overnight *R. solanacearum* culture was diluted to an OD_600_ of 0.05 in B medium supplemented with hydroxycoumarins (50 mg/L umbelliferone, 50 mg/L esculetin and 25 mg/L daphnetin) and incubated at 30 °C for 12 h. The treated bacterial cells were collected after centrifugation at 6000 rpm, fixed with 2.5% glutaraldehyde, post-fixed with 1% aqueous OsO_4_ (Fluka, Los Angeles, CA, USA) and washed with 0.1 M, PH 7.0 phosphate buffers. Thin sections containing the cells were placed on copper grids and observed under TEM microscope (FEI, Brno, Czech Republic).

### 3.9. RNA Isolation and Quantitative Real-Time RT-PCR

The effect of coumarins on the expression of virulence-associated genes of *R. solanacearum* was evaluated as previously reported [41]. Briefly, overnight-cultured *R. solanacearum* suspension adjusted to OD_600_ = 1.0 was inoculated in fresh B medium with coumarins or DMSO (the IC_50_ of coumarins against *R. solanacearum* after incubated for 12 h was listed in Table 3), then incubated on a shaker at 180 rpm and 30 °C for 6 h. The treated bacterial cells were collected after centrifugation. Total RNA was isolated using TRNzol reagent (Tiangen Biotech Co. Ltd., Beijing, China). qRT-PCR was used to evaluate the normalized expression of flagellar genes (*fli*A, *flh*C and *flh*D), and virulence-associated genes (*Prh*A, *Hrp*G, *Phc*S, *Phc*A, *Vsr*C, and *Eps*E) [40,41]. The primers of the tested genes used in this study were list in Table S2 and the housekeeping gene *Ser*C was used as the control [49]. qRT-PCR was performed on the CFX96 Manager (Bio-Rad) using an Sso Fast™ EvaGreen^®^ Supermix (Bio-Rad, Hercules, CA, USA). All assays were carried out at least three times in biological repeats.

## 4. Conclusions

In conclusion, this study evaluated the antibacterial activity of 18 plant-derived coumarins against *R. solanacearum*. Moreover, this is the first study to investigate the antibacterial activity and mechanism of action of hydroxycoumarins (umbelliferone, esculetin, and daphnetin) against *R. solanacearum*. Among the tested compounds, daphnetin exhibited the best antibacterial activity, followed by esculetin and umbelliferone, which showed that dihydroxylation in positions C-7 and C-8 of the coumarin structure enhanced the bactericidal activity against *R. solanacearum*. The mechanism of antibacterial action of hydroxycoumarins could be at least partially attributed to the destruction of the cell membrane. Hydroxycoumarins also significantly reduced the biofilm formation and repressed the swimming motility of *R. solanacearum*, resulting in weaker pathogenicity. The molecular mechanism of biofilm inhibition was shown to be related to the down-regulation of the regulating and structural flagellar genes (*fli*A and *flh*C). In addition, coumarins repressed the expression of the type III secretion system genes such as *HrpG* and *PrhA*. Future studies are expected to assess the antibacterial activity of hydroxycoumarins against other important agricultural pathogenic bacteria, and to design and synthesize coumarins which have better biological activity based on the hydroxycoumarins model. These investigations on the antibacterial and antibiofilm activities of hydroxycoumarins have shown that daphnetin and esculetin have the potential to be effective bacterial wilt control agents and applied as a new strategy to control plant bacterial diseases.

## Figures and Tables

**Table 1:** The antibacterial activity of coumarins against *R. solanacearum*.

Number	Compound	Antibacterial Rate (%) (Mean ± SD) ^a^	
		10 mg/L	100 mg/L
**1**	Coumarin	3.9 ± 1.1 *	50.3 ± 3.3 *
**2**	Scopoletin	1.5 ± 7.6	32.6 ± 4.9 *
**3**	Scoparone	1.0 ± 7.0	17.3 ± 2.9 *
**4**	Isofraxidin	2.5 ± 3.2 *	0.7 ± 1.4 *
**5**	3-Acetyl-2*H*-chromen-2-one	6.3 ± 4.7	10.7 ± 2.7 *
**6**	4-Methoxycoumarin	7.3 ± 1.9 *	54.1 ± 4.2 *
**7**	Osthole	0.2 ± 1.9 *	3.1 ± 3.3 *
**8**	4-Hydroxycoumarin	2.1 ± 5.7 *	4.9 ± 6.6 *
**9**	Umbelliferone	7.3 ± 3.0	59.7 ± 3.8
**10**	Esculetin	9.2 ± 2.8	71.4 ± 2.1 *
**11**	Daphnetin	13.3 ± 3.0	97.4 ± 0.7 *
**12**	Psoralen	13.1 ± 0.8	57.1 ± 8.8
**13**	Xanthotol	8.5 ± 5.4	80.1 ± 2.5 *
**14**	Xanthotoxin	6.3 ± 5.0	36.3 ± 5.4 *
**15**	Imperatorin	6.5 ± 2.9 *	19.1 ± 2.8 *
**16**	Isoimperatorin	4.1 ± 2.0 *	23.9 ± 6.3 *
**17**	Bergapten	4.7 ± 2.4 *	21.9 ± 1.6 *
**18**	Isopimpinellin	0.3 ± 1.6 *	22.5 ± 5.1 *
Positive Control	Thiodiazole Copper	12.2 ± 1.7	63.6 ± 2.9

^a^: The experiment was repeated in triplicates. Asterisks indicate statistically significant differences in antibacterial activity against *R. solanacearum* compared with thiodiazole copper treatment. (* indicates *p* < 0.05, Student’s *t* test).

**Table 2:** The minimum inhibitory concentrations (MICs) and minimum bactericidal concentrations (MBCs) of hydroxycoumarins against *R. solanacearum* in the 96-well polystyrene microtiter plates.

Coumarins	MIC (mg/L)	MBC (mg/L)
Coumarin	384	512
Umbelliferone	256	384
Esculetin	192	192
Daphnetin	64	64

Each experiment was repeated in three times.

**Table 3:** The IC_50_s of coumarins against *R. solanacearum.*

Coumarins	12 h			24 h		
	Toxicity Regression Equations	IC_50_ (mg/L)	R Value	Toxicity Regression Equations	IC_50_ (mg/L)	R Value
Coumarin	Y = 1.0293X + 3.1841	57.11	0.9330	Y = 1.2294X + 2.1747	198.64	0.9783
Umbelliferone	Y = 1.501X + 2.6435	37.15	0.9820	Y = 1.8022X + 1.4204	96.88	0.9683
Esculetin	Y = 2.0185X + 2.1871	24.75	0.9712	Y = 2.1808X + 1.0057	67.85	0.9807
Daphnetin	Y = 2.0489X + 3.0719	8.73	0.9863	Y = 3.2992X + 0.7286	23.98	0.9809

## Supplementary Materials

Supplementary materials can be accessed at: http://www.mdpi.com/1420-3049/21/4/468/s1.
