# Supplementary material for: New Insights into the Antibacterial Activity of Hydroxycoumarins against Ralstonia solanacearum
Source: Molecules. 2016 Apr 8;21(4):468. doi: 10.3390/molecules21040468 (PMC6273506; doi:10.3390/molecules21040468)
Supplement: Supplementary file 1 [file molecules-21-00468-s001.pdf]

Supplementary Materials: New Insights into the Antibacterial Activity of Hydroxycoumarins against *Ralstonia solanacearum*

Liang Yang, Wei Ding, Yuquan Xu, Dousheng Wu, Shili Li, Juanni Chen and Bing Guo

TableS1. The antibacterial activity of plant-derived compounds against *R. solanacearum*.

Number	Compound (100 mg/L)	Antibacterial Rate (%) (Mean ± SD)
**1**	Glycyrrhetinic acid	2.4 ± 4.5 *
**2**	Emodin	22.1 ± 1.3 *
**3**	Puerarin	4.5 ± 2.6 *
**4**	Eugenol	37.7 ± 5.4 *
**5**	Quercetin	21.8 ± 3.5 *
**6**	Rutin	41.2 ± 4.6 *
**7**	Betaine	12.5 ± 2.4 *
**8**	Theophylline	0.5 ± 6.5 *
**9**	3,4-Dihydroxybenzoic acid	17.5 ± 3.6 *
**10**	Ursodeoxycholic acid	0.3 ± 7.1 *
**11**	Nicotinic Acid	1.6 ± 4.3 *
**12**	Tea polyphenol	50.2 ± 8.5
**13**	Coumarin	55.6 ± 2.8
**14**	Umbelliferone	60.7 ± 5.2
**15**	Clove oil	32.5 ± 1.8 *
Positive Control	Thiadiazole Copper	54.2 ± 4.5

The experiment was repeated in triplicates. Asterisks indicate statistically significant differences in antibacterial activity against *R. solanacearum* compared with Thiediazole Copper treatment. (* indicates $p < 0.05$, Student's t test).

Table S2. Primers used in this study.

Name	Sequence (5′ to 3′)	Source
Serc-F	CCCACCTACGCCATCTATGT	[1]
Serc-R	TTGAGGAAGAACGGCACATT	[1]
*fli*A-F	TCGGAGACGCCCGAGACCTT	[2]
*fli*A-R	TCGGAGACGCCCGAGACCTT	
*flh*C-F	CTTCCTGAACATCTACCGTTTC	[2]
*flh*C-R	GAGTGATCGACAGCACCTCTT	
*flh*D-F	CTGGCTGACATCCTGCTGA	[2]
*flh*D-R	CGACATTTGCGATTGCTG	
PrhA-F	GGTAGATCAGGCCGTTCGT	[1]
PrhA-R	GACGAGATCGCTGTCATCAA	[1]
HrpG-F	GTCTTCACGGTCTGCGAACT	[1]
HrpG-R	ATTGACCTCCAATCCATCCA	[1]
PhcS-F	CCAGCTGAAAGAGGAACTGG	[1]
PhcS-R	AGAAGTTGACGGGGTTGTTG	[1]
PhcA-F	TTGTAGGTCTCGCACACCAG	[1]
PhcA-R	GCTCGCTCGATCAGTACCTC	[1]
VsrC-F	ACCACCCTCTCGCCTTATCT	This study
VsrC-R	ACAGCCAGACATCCAGCAG	This study
EpsE-F	CTGGATAAAGCCACGCAAAG	[1]
EpsE-R	CAGTGGTACATCGCCATCAC	[1]

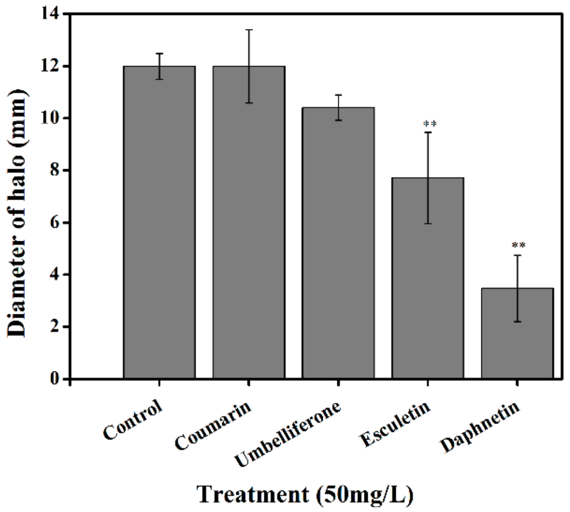

Figure S1. The effect of coumarins on the swimming motility of *R. solanacearum*. The halo diameter was quantified on the semi-solid agar plates after treatment with different coumarins. The experiment was performed in duplicates, and each replicate contained at least three plates for each coumarin. (** indicated $p < 0.05$).

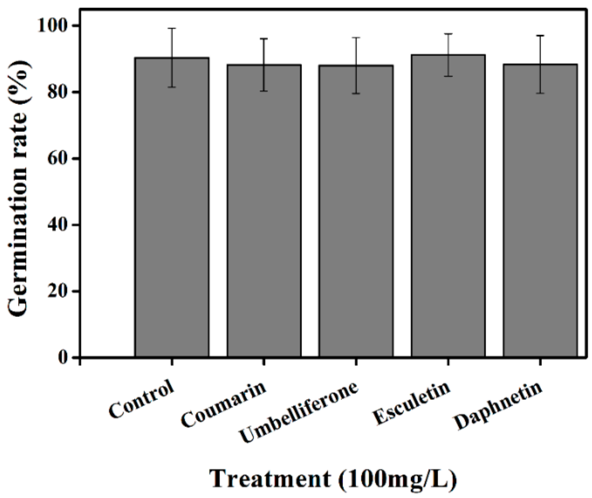

Figure S2. The effect of coumarins on the germination rate of tobacco seed. The germination rate of tobacco seed was quantified on the MS medium after treatment with coumarins and cultured for 3–5 days. The results were observed in two independent experiments, and each replicate contained at least five plates for each coumarin.

References

1. Wu, D.; Ding, W.; Zhang, Y.; Liu, X.; Yang, L. Oleanolic acid induces the type III secretion system of *Ralstonia solanacearum*. *Front. Microbiol.* **2015**, *6*, 1466.
2. Zhang, L.; Xu, J.; Xu, J.; Zhang, H.; He, L.; Feng, J. TssB is essential for virulence and required for type VI secretion system in *Ralstonia solanacearum*. *Microb. Pathog.* **2014**, *74*, 1–7.
